# Insights into attention and memory difficulties in post-COVID syndrome using standardized neuropsychological tests and experimental cognitive tasks

**DOI:** 10.1038/s41598-024-54613-9

**Published:** 2024-02-22

**Authors:** Sandra Arbula, Elisabetta Pisanu, Giulia Bellavita, Alina Menichelli, Alberta Lunardelli, Giovanni Furlanis, Paolo Manganotti, Stefano Cappa, Raffaella Rumiati

**Affiliations:** 1 Neuroscience Area, International School for Advanced Studies (SISSA), Via Bonomea 265, 34136 Trieste, Italy; 2 Neurology Unit, Department of Medicine, Surgery and Health Sciences, Trieste University Hospital ASUGI, Trieste, Italy; 3 Rehabilitation Unit, Department of Medicine, Surgery and Health Sciences, Trieste University Hospital ASUGI, Trieste, Italy; 4 Scuola Universitaria Superiore IUSS, Pavia, Italy; 5 IRCCS Mondino Foundation, Pavia, Italy; 6 Università Degli Studi di Roma “Tor Vergata”, Rome, Italy

**Keywords:** Neurological disorders, Human behaviour, Cognitive neuroscience

## Abstract

The COVID-19 pandemic has given rise to post-acute cognitive symptoms, often described as ‘brain fog’. To comprehensively grasp the extent of these issues, we conducted a study integrating traditional neuropsychological assessments with experimental cognitive tasks targeting attention control, working memory, and long-term memory, three cognitive domains most commonly associated with ‘brain fog’. We enrolled 33 post-COVID patients, all self-reporting cognitive difficulties, and a matched control group (N = 27) for cognitive and psychological assessments. Our findings revealed significant attention deficits in post-COVID patients across both neuropsychological measurements and experimental cognitive tasks, evidencing reduced performance in tasks involving interference resolution and selective and sustained attention. Mild executive function and naming impairments also emerged from the neuropsychological assessment. Notably, 61% of patients reported significant prospective memory failures in daily life, aligning with our recruitment focus. Furthermore, our patient group showed significant alterations in the psycho-affective domain, indicating a complex interplay between cognitive and psychological factors, which could point to a non-cognitive determinant of subjectively experienced cognitive changes following COVID-19. In summary, our study offers valuable insights into attention challenges faced by individuals recovering from COVID-19, stressing the importance of comprehensive cognitive and psycho-affective evaluations for supporting post-COVID individuals.

## Introduction

The emergence of post-acute cognitive symptoms of COVID-19 has received significant attention in recent research. Some of the most frequently reported sequelae of COVID-19 involve symptoms such as headaches, fatigue, anxiety, depression, and cognitive impairments, colloquially referred to as ‘brain fog’^1^. The ‘brain fog’ phenomenon is characterized by a constellation of cognitive difficulties, including slow thinking, forgetfulness, reduced concentration, and difficulty in maintaining focus^2,3^. Epidemiological studies indicate that up to approximately 20% of COVID survivors experience objective cognitive impairment^4^, with assessments typically relying on standardized neuropsychological tests. However, to comprehensively understand the scope and nature of cognitive deficits associated with COVID-19, it is essential to employ a multifaceted approach. In this study, we sought to enhance our understanding of the cognitive repercussions of COVID-19 by complementing traditional neuropsychological assessments with a battery of experimental cognitive tasks designed to thoroughly probe aspects of attention control, working memory, and long-term memory.

Given the multifaceted nature of cognitive difficulties after COVID-19, employing a diverse set of measures can provide a more nuanced perspective on the cognitive challenges faced by post-COVID patients. While traditional neuropsychological tests have long been considered the gold standard for assessing cognitive impairment, they may not capture subtle deficits in the specific cognitive domains affected by ‘brain fog’. Our working hypothesis was that experimental cognitive tasks designed to evaluate these cognitive domains may offer greater sensitivity in detecting impairments reported by patients with ‘brain fog’. By including tasks that target attention control, working memory, and long-term memory, we aimed to provide a more discerning and comprehensive evaluation of cognitive functioning in post-COVID individuals and compare it to standard neuropsychological assessments.

We have focused on several different types of working memory and attention control measures, as derived from the extensive toolbox of tasks tailored for assessing these cognitive domains in young adults^5^. Specifically, we incorporated three attention control tasks designed to capture individual differences, a facet frequently compromised in cognitive tasks primarily geared toward comparing experimental conditions. These attention tasks are held to tap interference resolution, inhibitory control and selective attention components. In addition, we included two working memory tasks, focusing on verbal and visuo-spatial aspects, along with a long-term memory task that examined both relational and item-specific encoding. This comprehensive approach allowed us to delve deeper into the frequently reported attention and memory difficulties observed in post-COVID patients. In addition to these specific tasks, we also integrated an extensive neuropsychological battery into our study design, which allowed us to make direct comparisons between their performances on experimental cognitive measures and established standard neuropsychological test scores. This approach addresses a significant gap in the literature, as shown by a recently published meta-analysis on cognitive impairments after COVID-19^6^, which highlighted very few studies that gathered data from both types of assessment. This combination of assessments is vital in shedding light on the cognitive profile of post-COVID patients, especially those with low to mild cognitive symptoms.

Another distinctive aspect of our study is the inclusion of a healthy control group. Unlike many previously published studies on post-COVID cognitive impairments (see Velichkovsky et al.^6^ for a recent review), we compared patients’ performance on measures derived from the experimental cognitive tasks to a well-matched control group, thus allowing us to discern specific cognitive deficits linked to COVID-19. This comparative approach enhanced the reliability of our findings, providing valuable insights into the cognitive challenges post-COVID individuals face and their distinction from general cognitive function.

## Method

### Participants and procedure

Thirty-three patients (25 female) with a mean age of 54.1 (SD = 6.9) and an age range of 40 to 67 were enrolled on a voluntary basis to participate in this study. Patients were admitted to the post-COVID neurological unit between January 2021 and March 2022. They reported cognitive difficulties following COVID-19 infection. We excluded patients who suffered from moderate-to-severe COVID-19 disease, defined as patients positive for SARS-CoV-2 with clinical and radiographic evidence of lower respiratory tract disease and hospitalized for respiratory failure due to COVID-19. On average, the patients were tested 8.3 months (SD = 4.2) after experiencing their first symptom onset. Two testing sessions were conducted. The first session consisted of a comprehensive battery of neuropsychological tests, while the second session involved experimental cognitive tasks, assessments of psychological well-being, and fluid intelligence. Two patients did not complete the neuropsychological assessment.

A healthy control group (N = 27) was included, evaluated solely on the cognitive tasks battery without published normative data for the relevant age group. We ensured that, based on their self-reported knowledge, they had not contracted the COVID-19 virus. They were matched for age (patients: mean = 54 (SD = 7), controls: mean = 57 (SD = 6.1); H(1) = 3.72, p = 0.054), gender (patients: F = 25, M = 8; controls: F = 17, M = 10; χ^2^ = 0.63, p-value = 0.427), and education (patients: mean = 13.55 (SD = 3.13), controls: mean = 14.15 (SD = 3.12); H(1) = 0.27, p = 0.602). A sensitivity power analysis conducted in G*Power^7^ for MANOVA interactions assessing the experimental cognitive task measures revealed that our sample had a power of 80% to detect a minimum effect size of *f* ^2^ = 0.075 for 2 groups and 10 response variables. Supplementary Table S1 provides all demographic data. Prior to participating in the testing sessions, all participants provided written informed consent. The study received approval from the Local Ethics Committee CEUR (Comitato Etico Unico Regionale, FVG, Italy) and adhered to the guidelines outlined in the Declaration of Helsinki.

### Materials

#### Neuropsychological assessment

All patients underwent a comprehensive standard neuropsychological assessment that encompassed the primary cognitive domains, including language (verbal fluency and naming), short-term and working memory, verbal and visuo-spatial long-term memory, attention, and executive functions. Additionally, self-rated memory failures were assessed through the Prospective and Retrospective Memory Questionnaire^8^. Detailed information regarding the specific tests used and their respective specifications can be found in Supplementary Table S1, which presents the complete set of neuropsychological data. Furthermore, during the second session, both patients and healthy controls were evaluated for their psychological well-being using the State-Trait Anxiety Inventory (STAI-Y)^9^, the Beck's Depression Inventory for primary care (BDI-PC)^10^ and the post-traumatic stress disorder risk related to COVID-19^11^. Additionally, the assessment of fluid intelligence was conducted using the abbreviated Raven Progressive Matrices (RPM) test^12^.

#### Experimental attention and memory tasks

The experimental battery of tasks was adapted from an extensive set of tasks designed to assess working memory and attention control in healthy young adults, available at https://englelab.gatech.edu/taskdownloads. Additionally, a long-term memory task was included to assess single item and relational encoding, available at https://cntracs.ucdavis.edu/tasks. A more detailed description of the tasks and the dependent measures for each task are reported in Supplementary materials.

##### Working memory capacity

To evaluate working memory capacity, we employed shortened versions of the Operation and Symmetry span tasks^13^. In the Operation Span task, participants were presented with a series of arithmetic operations and asked to judge their validity, while in the Symmetry Span task, participants were presented with matrices of black and white squares and had to determine their vertical axis symmetry. After each arithmetic operation or matrix presentation, participants were required to remember a specific element (a letter in the Operation Span task and a red square in the Symmetry Span task) for later recall.

#### Attention control

Three tasks were selected from the Toolbox of Attention Control Measures^5^.

##### Antisaccade task^14,15^

Participants were presented with an asterisk that appeared to the left or right of the center, followed by a letter (Q or O) displayed on the opposite side of the screen. The participants' task was to disregard the asterisk and shift their attention to the opposite side of the screen to identify the target letter.

##### Selective visual arrays^16,17^

Participants viewed a display of blue and red rectangles with different orientations and were instructed to attend to either the red or blue rectangles. The target rectangles reappeared (red or blue) with one of the rectangles indicated by a white dot, which randomly changed its orientation on half of the trials. Participants were required to indicate whether the rectangle with the white dot had changed its orientation from the initial presentation.

##### Adaptive Flanker task (deadline version)^5^

Participants were shown an arrow at the center of the screen pointing left or right, along with two surrounding arrows on each side that either matched the direction of the central arrow (congruent trial: e.g., ⟵⟵⟵⟵⟵) or pointed in the opposite direction (incongruent trial: e.g., ⟶⟶⟵⟶⟶). The participants were instructed to indicate the direction of the central arrow.

##### Long-term memory

The stimuli used in the Relational and Item Specific Encoding task^18^ consisted of visual object representations selected from a standardized corpus of color photographs. Participants performed two incidental encoding tasks. In the item-specific encoding task, participants were instructed to indicate whether the objects were "living" using a two-button yes/no response. In the Relational Encoding task, participants were instructed to indicate whether one object could fit inside the other using a two-button yes/no response. After the encoding phase, two retrieval tasks were administered. In the item recognition task, participants were prompted to indicate whether each item was "old" or "new". In the associative recognition task participants were prompted to indicate whether the items in each pair had been presented "together".

### Data analysis

Patients’ scores from the neuropsychological assessment were normalized by z-transforming them with respect to the mean and standard deviation of the normative sample, divided for age, sex and/or education, based on the data provided in the referenced test manuals (Supplementary Table S1). The average group z scores are reported in Table 1. Additionally, for each neuropsychological test score, we performed a pairwise comparison between the patients’ scores and the normative sample datasets, which were simulated using the ‘rnorm’ function in R using the mean, SD and sample size as provided in the referenced test manuals. Patients’ scores were corrected for age, education and gender before the comparison if an effect of these factors was observed in the normative sample dataset. When variance between the two groups differed, the Welch two-sample t-test was employed; otherwise, the two-sample t-test was used.

**Table 1:** Neuropsychological scores from post-COVID patient sample normalized with respect to the mean and standard deviation of the normative sample.

Test	Subtest	Median	Mean	SD	% < − 1.5	% < − 2.5	*t*	*df*	*p*	Cohen’s d
MoCA		0.33	0.21	0.66	3	0	− 1.239	39.83	0.223	− 0.185
PRMQ	Prospective	**− 2.10**	**− 1.82**	1.27	**61**	**23**	**− 8.260**	**33.41**	**1.402E−09**	**− 1.635**
	Retrospective	**− 1.57**	**− 1.21**	1.24	**42**	**10**	**− 5.099**	**33.06**	**1.37E−05**	**− 1.036**
	Total	**− 1.87**	**− 1.64**	1.32	**48**	**16**	**− 6.884**	**32.97**	**7.361E−08**	**− 1.408**
Digit Span	Forward	0.34	0.54	1.13	0	0	− 2.502	51.83	0.016	− 0.346
	Backward	0.44	0.44	1.02	3	0	− 3.489	393	0.001	− 0.634
Word list	Immediate recall	0.02	0.24	1.19	6	0	− 1.128	42.04	0.266	− 0.205
	Delayed recall	0.22	0.31	1.28	6	0	− 1.033	280	0.302	− 0.191
	Non recalled	0.39	0.31	0.87	6	0	0.428	46.20	0.671	0.072
	Recognition	0.10	− 0.25	0.91	10	6	1.671	52.63	0.101	0.261
TMT	TMT-A	0.01	− 0.40	1.39	13	6	0.341	62.04	0.734	0.043
	TMT-B	− 0.46	**− 1.02**	1.82	**19**	**16**	**− 2.745**	**61.45**	**0.008**	**− 0.348**
	TMT-B-A	− 0.65	**− 1.00**	1.37	**19**	**16**	**− 2.358**	**57.38**	**0.022**	**− 0.309**
SDMT		− 0.02	− 0.11	0.61	3	0	1.358	43.68	0.181	0.205
Stroop	Congruent	− 0.41	− 0.44	0.68	6	0	**3.863**	**52.60**	**3.09E−04**	**0.658**
	Incongruent	− 0.09	− 0.22	0.69	6	0	2.160	54.35	0.04	0.363
PASAT		− 0.32	− 0.31	1.27	16	6	**2.982**	**177**	**3.27E−03**	**0.575**
MFPT	Unique designs	− 0.70	− 0.57	0.98	16	0	**3.518**	**363**	**4.90E−04**	**0.642**
	Strategies	− 0.41	− 0.41	0.27	0	0	**4.728**	**172.61**	**4.70E−06**	**0.454**
	Errors	0.23	− 0.12	1.86	6	6	− 0.604	33.98	0.550	− 0.132
Fluency	Phonemic	0.20	0.17	1.01	3	0	− 0.438	321	0.661	− 0.081
	Semantic	1.20	1.18	1.45	0	0	− 4.332	34.21	1.23E−04	− 0.897
Naming		**− 1.22**	**− 1.06**	1.37	**16**	**13**	**2.962**	**137**	**3.61E−03**	**0.590**
COVID PTSD		− 0.20	− 0.31	0.93	13	0	**− 2.192**	**2317**	**0.028**	**− 0.384**

Evidenced in bold are the average scores below 1 SD from the normative sample. For the pairwise Group comparison, significant p values (< .031 false discovery rate multiple comparison correction) are evidenced in bold.

*MoCA* montreal cognitive assessment, PRMQ = Prospective and Retrospective Memory Questionnaire; TMT = Trail Making Test; SDMT = Symbol Digit Modalities Test; PASAT = Paced Auditory Serial Addition Task; MFPT = Modified Five-Point Test; PTSD = Post-traumatic Stress Disorder.

Scores from the BDI, STAI-Y and Raven were compared to the control group by means of the nonparametric Kruskal‒Wallis rank sum test, since they differed significantly from the normal distribution, as assessed by a Shapiro‒Wilk test.

In the attention control tasks, trials with anticipated responses (i.e., RTs < 150 ms) were excluded from all analyses. Ten performance measures from the attention and memory tasks were included in the MANOVA as dependent variables, with Group (patients vs. controls) as a between-subjects factor. To improve normality, all dependent variables were transformed (‘BestNormalize’ R function^19^), and multivariate normality was assessed through the Henze-Zirkler test (‘MVN’ R function^20^). Before proceeding with the analysis, the Mahalanobis distance was employed to identify potential multivariate outliers, and the homogeneity of covariance matrices was tested across groups using Box's M test (‘rstatix’ R package, https://rpkgs.datanovia.com/rstatix/). A significant MANOVA result was followed by a descriptive discriminant analysis (DDA) to determine which performance measures contributed most to group differences. In particular, the structure coefficients, indicating the correlation between the observed (i.e., dependent) and the composite variable (i.e., MANOVA output variable maximizing group differences) with absolute values >|.32| were considered relevant^21^.

As for the neuropsychological scores, the experimental cognitive task scores were also normalized by z-transforming them with respect to the mean and standard deviation of the control sample, and pairwise comparison were performed between the two groups by means of two sample t-tests, adapted for differences in variance when necessary.

## Results

For the neuropsychological assessment, post-COVID patients showed a significantly worse performance with respect the normative sample on multiple tests mainly assessing attention and executive functions (TMT-B, Stroop, PASAT, MFPT), and naming abilities (Table 1). The average z scores 1 SD lower with respect to the normative sample were observed on the TMT-B and naming tests (Table 1). The Prospective and Retrospective Memory Questionnaire (PRMQ) showed a high incidence of subjective memory complaints: post-COVID patients differed significantly and scored on average below 1 SD from the normative sample for both prospective and retrospective scales (Table 1). Post-COVID patient also showed significantly higher scores for the post-traumatic stress disorder risk related to COVID-19 (Table 1).

**Figure 1:**
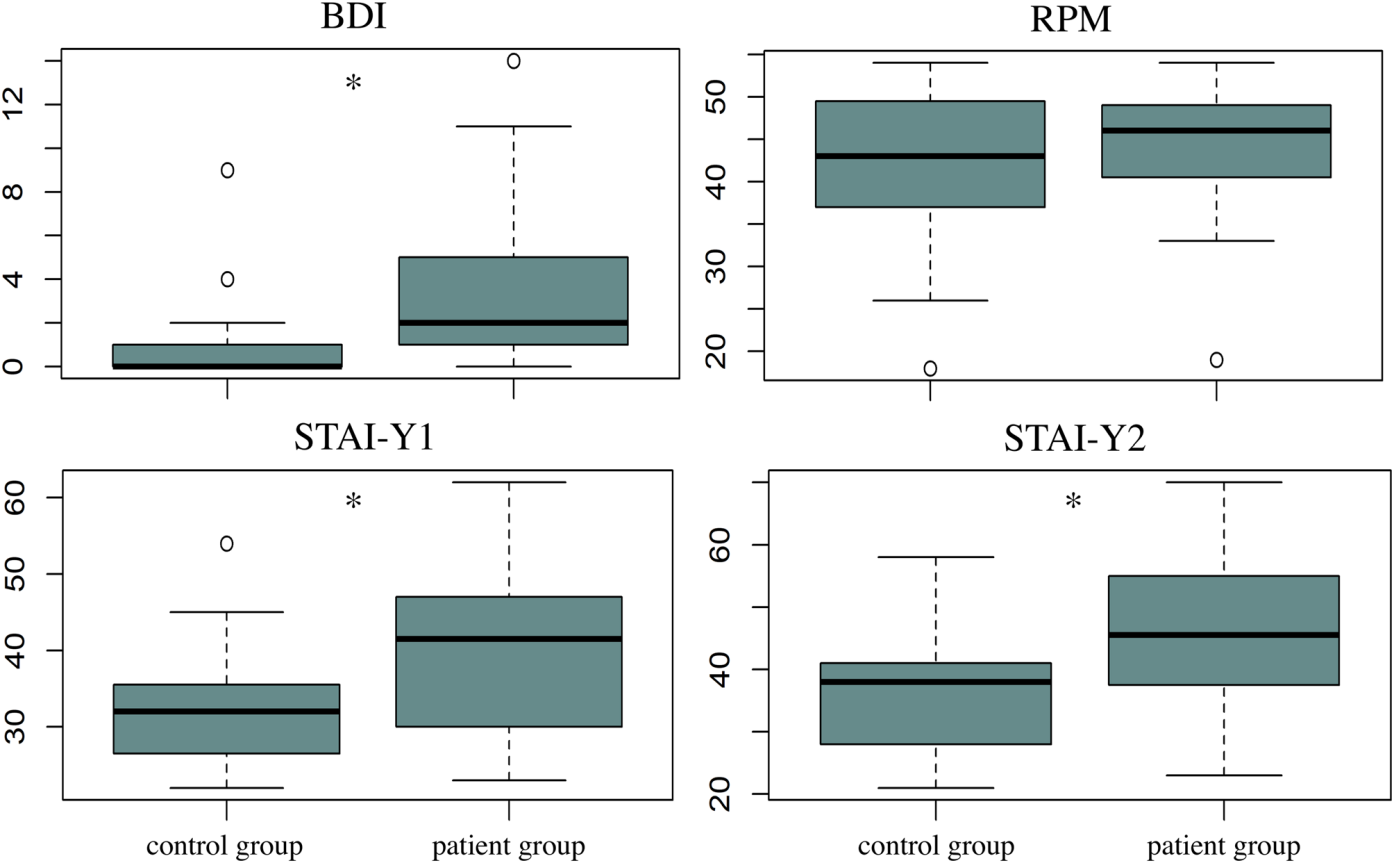
Psycho-affective and general intelligence scores. Boxplot distribution of depression (BDI), state (STAI-Y1) and trait (STAI-Y2) anxiety and general intelligence (RPM) data for the control and patient groups. The asterisks denote significant group differences.

State and trait anxiety measures and depression scores were significantly higher in the patient group than in the control group (STAI-Y1 state: H(1) = 6.55, p = 0.01; STAI-Y2 trait: H(1) = 8.42, p = 0.004; BDI: H(1) = 12.23, p = 0.0005) (Fig. 1). The two groups did not differ in terms of general intelligence (RPM: H(1) = 0.33, p = 0.57) (Fig. 1).

**Figure 2:**
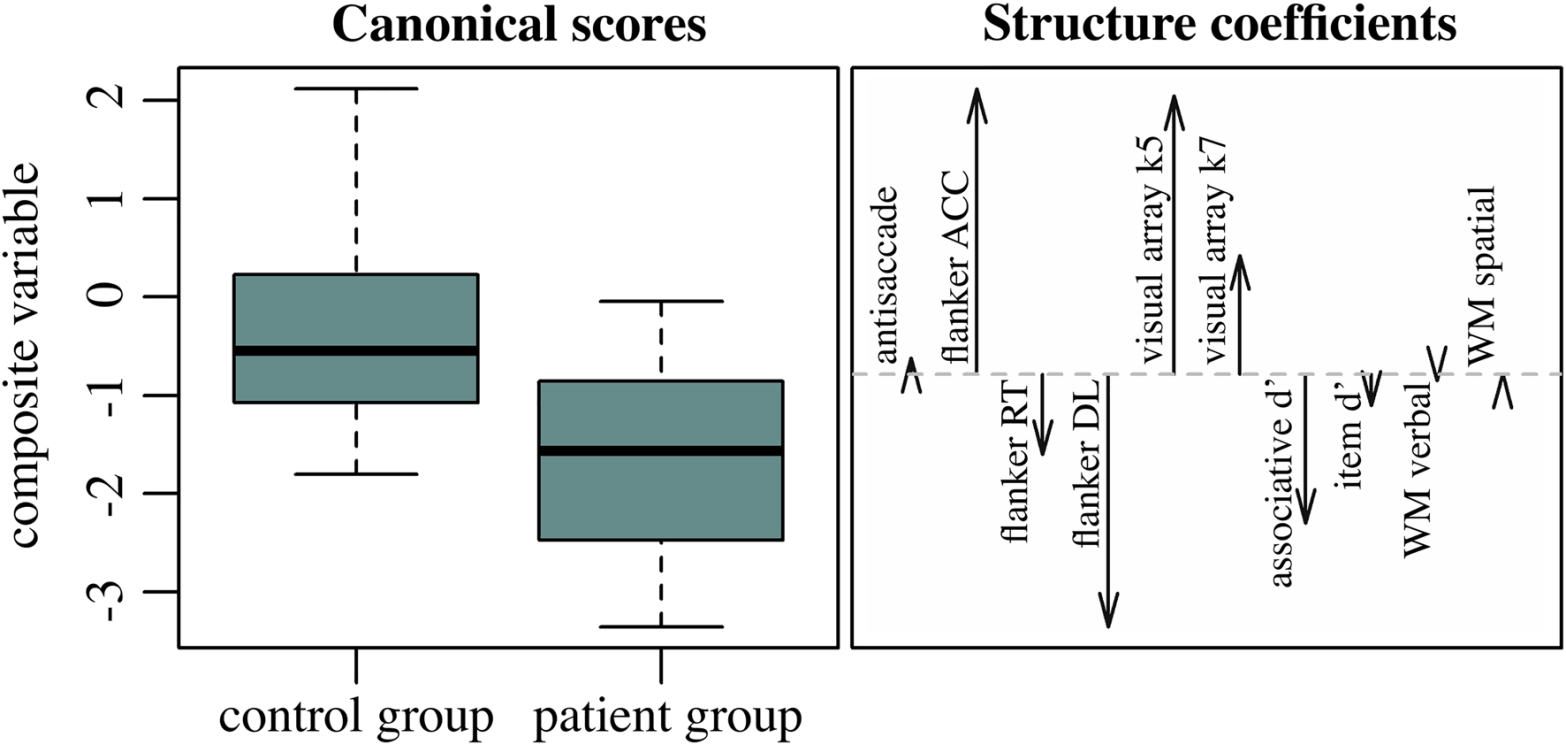
MANOVA and DDA results. Boxplot distribution of the MANOVA composite variable for the control and patient groups and structure coefficients size and direction from the DDA for each dependent variable.

The MANOVA for attention and memory performance measures from the experimental cognitive tasks battery revealed a significant Group effect (Pillai's Trace = 0.278, F(9, 50) = 2.14, p = 0.043, *η*^2^ = 0.28). According to the structure coefficients from the DDA (Table 2, Fig. 2), performance measures from two attention tasks, Flanker and Visual Arrays, were found to contribute mostly to discriminating between patients and controls. The assumptions for multivariate normality and for homogeneity of covariance were met (HZ = 0.98, p = 0.313; Box’s M = 68.85, p = 0.099). No patients were identified as outliers. When assessing group differences in a pairwise manner, patients performed worse with respect to the control group on both Flanker and Visual Array tasks, although these differences did not survive multiple comparison correction (Table 3).

**Table 2:** Discriminant function structure coefficients for each dependent variable and group centroids for the MANOVA composite variable, with group difference effect size (Cohen’s d).

Outcome variable	Structure coefficients	
Antisaccade accuracy	0.032	
Flanker accuracy	0.562	
Flanker RTs	− 0.157	
Flanker deadline	− 0.496	
Visual Array k5	0.548	
Visual Array k7	0.234	
Association recognition d'	− 0.292	
Item recognition d'	− 0.061	
WM verbal	− 0.012	
WM spatial	0.001	

Group	Composite variable	
	Centroids [95% CI]	Cohen’s d [95% CI]
Controls	0.71 [0.29, 1.13]	− 1.31 [− 1.87, 0.74]
Patients	− 0.58 [− 0.91, − 0.24]

**Table 3:** Experimental cognitive tasks scores from the post-COVID patient sample normalized with respect to the mean and standard deviation of the control sample.

Test	Measure	Median	Mean	SD	% < − 1.5	% < − 2.5	*t*	*df*	*p*	Cohen’s d
Antisaccade	Accuracy	0.18	0.00	0.88	6	3	0.135	58.00	0.893	0.035
Flanker	Accuracy	− 0.54	− 0.77	1.33	30	12	2.412	51.30	0.019	0.631
	RTs	− 0.25	− 0.67	1.72	15	9	− 2.139	58.00	0.037	− 0.555
	Deadline	− 0.05	− 0.39	1.61	15	9	− 0.672	57.74	0.504	− 0.172
Visual Array	k5	− 0.93	− 0.83	1.33	24	15	2.428	57.93	0.018	0.625
	k7	− 0.22	− 0.37	1.39	21	3	1.002	58	0.321	0.257
Long term memory	Association recognition d’	0.76	0.49	1.37	6	3	− 1.259	57.84	0.213	− 0.323
	Item recognition d’	0.12	0.07	0.93	6	3	− 0.250	53	0.804	− 0.065
Working memory	Verbal	− 0.01	− 0.05	1.14	0	0	− 0.052	57.92	0.958	− 0.014
	Spatial	− 0.15	− 0.04	1.08	0	0	0.005	57.24	0.996	0.001

For the pairwise Group comparison, none of the results survived the false discovery rate multiple comparison correction.

## Discussion

Our study aimed to assess cognitive aspects of post-COVID syndrome in order to investigate more thoroughly the subjective attention and memory deficits often reported in individuals recovering from COVID-19^22–26^. The results of the MANOVA showed a significant Group effect, suggesting a comprehensive attention and memory deficit in post-COVID patients with respect to the control group. The post-hoc discriminant analysis revealed that performance measures from two attention tasks, Flanker and Visual Arrays, played a significant role in discriminating between patients and controls. This result was partially confirmed by the pairwise comparisons, although the significance threshold did not survive multiple comparison correction (Table 3). The observed impairments in these tasks assessing interference resolution and selective attention align with the reported difficulties in concentration and focus, often described as part of ‘brain fog’^2,27^. This suggests that attentional deficits may be a core feature of cognitive dysfunction in post-COVID individuals, as supported by different systematic reviews conducted in the last two years^6,28–30^. While the specific memory tasks did not independently reach significance in our MANOVA, their contribution to the overall cognitive profile was still relevant. Memory deficits have also been consistently reported in the above-cited systematic reviews, and our results align with this pattern.

However, contrary to our hypothesis, experimental cognitive tasks did not offer greater sensitivity in detecting impairments reported by patients with ‘brain fog’ with respect to the standardized neuropsychological assessment. In particular, post-COVID patients showed lower performance across multiple tasks tapping attention, executive functions and language. However, we observed some differences in the results based on the type of analysis that was adopted. While pairwise group comparisons evidenced multiple tasks in which patients performed below the average performance of the normative sample, the standardized z-scores at the subject level revealed only mild impairments (i.e., below 1 SD from the normative sample) across fewer tasks (Table 1). This discrepancy in the results suggests that even though post-COVID patients did show lower performance on different tasks, especially tapping attention and executive functions, their scores were not considered deficient when standardized with respect to the normative sample. Notably, 61% of post-COVID patients reported considerable prospective memory failures in everyday life, a finding in alignment with our patient recruitment strategy, which exclusively included individuals who self-reported cognitive difficulties following COVID-19 infection. This finding speaks in favor of a discrepancy between the subjective cognitive symptoms and objective cognitive impairments, which in some cases has been associated with ‘brain fog’ (e.g., in chronic fatigue syndrome, fibromyalgia and functional neurological disorder^31^).

Functional cognitive disorder is defined as a condition of cognitive symptoms with clear evidence of internal inconsistency (i.e., inconsistent performance over tasks from similar cognitive domains, observed performance during interviews, and/or real-life performance), not better explained by another disorder, and causing distress or impairment^32^. It is mainly characterized by memory and attention lapses, mental fatigue and word-finding difficulties, a similar symptomatology as ‘brain-fog’ in post-COVID patients. The primary pathophysiologic processes associated with functional neurological disorder involve alterations across multiple brain networks^33^, alterations that have also been observed in post-COVID patients^34^. A recent systematic review^35^ investigated the occurrence of functional neurological disorder in people with long COVID. The authors reported several studies that failed to observe a consistent objective impairment in long COVID patients, and other studies in which cognitive impairments were found mainly in hospitalized patients. Although the authors concluded that little evidence was found suggesting a contribution of functional cognitive symptoms to long COVID, they did observe that these symptoms were also rarely considered. Even though in our study we did not address this possible association specifically, our results seem to point in Teodoro and colleagues’^35^ direction, highlighting reduced attentional abilities as the focal impairment that might underlie the ‘brain fog’ symptomatology. Indeed, decreased attentional resources, along with heightened perception of cognitive effort, have been proposed as the mechanism underpinning cognitive difficulties across the functional cognitive disorder spectrum^31^. To test this hypothesis, future studies should assess both domains in post-COVID patients who present with cognitive complaints without a diagnosed neurological cause. A careful consideration of metacognitive abilities may also contribute to the understanding of functional cognitive disorders^36^, a wide group of conditions sharing multiple common aspects with the post-COVID “brain fog”^32^.

Furthermore, our patient group exhibited significantly higher levels of state and trait anxiety, as well as depression scores, than the control group. Anxiety and depression are among the most common COVID-19 sequelae, reported both in hospitalized and non-hospitalized patients^37,38^. Interestingly, subjective cognitive complaints after COVID-19 were found to be associated with anxiety and depression independently of objective neuropsychological status^39^, which raises the question of whether the reported cognitive alterations can be determined by psychiatric or psycho-affective conditions rather than substantial cognitive impairments. Our results partially support this hypothesis, since the post-COVID patients in our sample were selected based on subjective cognitive complaints, which did not translate into prominent cognitive impairment. This pattern, along with a higher incidence of anxiety and depression, is also reported in people suffering from functional cognitive disorder^31^. Still, it is important to underline that such a pattern might emerge more often in patients suffering from psycho-affective difficulties present already at the pre-COVID stage, and this important point should be addressed in future studies.

In conclusion, our study provides valuable insights into attention and memory difficulties among individuals recovering from COVID-19. However, it is important to acknowledge several limitations. Firstly, our study focused solely on patients reporting cognitive issues after COVID-19, and therefore, our findings are limited to this subgroup and may not extend to all COVID-19 patients. Moreover, without prior neuropsychological assessments, we cannot conclusively establish a causal link between COVID-19 and cognitive dysfunction, although subjectively all our patients reported a decline in cognitive performance after COVID-19. Secondly, the sensitivity power analysis we conducted, based on our sample size, could not encompass all the tests eventually employed. This limitation impacts the robustness and generalizability of our results. Finally, the higher incidence of anxiety and depression among post-COVID patients suggests potential psycho-affective contributions to reported cognitive alterations. However, our study did not comprehensively investigate the underlying neurobiological and psycho-affective mechanisms that would be necessary to draw this conclusion. In summary, our study underscores the need for comprehensive cognitive and psycho-affective assessments in post-COVID individuals and emphasizes the complex interplay between cognitive, psychological, and neurological factors, warranting further research for targeted interventions and support strategies.

### Supplementary Information

Supplementary Information 1.

Supplementary Information 2.

## Data Availability

The datasets analysed during the current study are available from the corresponding author on reasonable request.

## References

[1] Asadi-Pooya AA (2022). Long COVID syndrome-associated brain fog. J. Med. Virol..

[2] Jennings G, Monaghan A, Xue F, Duggan E, Romero-Ortuño R (2022). Comprehensive clinical characterisation of brain fog in adults reporting long COVID symptoms. J. Clin. Med..

[3] McWhirter L (2023). What is brain fog?. J. Neurol. Neurosurg. Psychiatry.

[4] Badenoch, J. B. *et al.* Persistent neuropsychiatric symptoms after COVID-19: A systematic review and meta-analysis. *Brain Commun.* **4**, 297 (2022). 10.1093/braincomms/fcab297 PMC8833580 35169700

[5] Draheim C, Tsukahara JS, Martin JD, Mashburn CA, Engle RW (2021). A toolbox approach to improving the measurement of attention control. J. Exp. Psychol. Gen..

[6] Velichkovsky, B. B., Razvaliaeva, A. Y., Khlebnikova, A. A., Manukyan, P. A. & Kasatkin, V. N. Attention and memory after COVID-19 as measured by neuropsychological tests: Systematic review and meta-analysis. *Acta Psychol. ***233**, 103838 (2023). 10.1016/j.actpsy.2023.103838 PMC9834202 36657196

[7] Faul, F., Erdfelder, E., Buchner, A. & Lang, A. G. Statistical power analyses using G*Power 31: Tests for correlation and regression analyses. *Behav. Res. Methods* **41**, 1149–1160 (2009). 10.3758/BRM.41.4.1149 19897823

[8] Crawford JR, Smith G, Maylor EA, Della Sala S, Logie RH (2003). The Prospective and Retrospective Memory Questionnaire (PRMQ): Normative data and latent structure in a large non-clinical sample. Memory.

[9] Spielberger, C. D., Gonzalez-Reigosa, F., Martinez-Urrutia, A., Natalicio, L. F. S. & Natalicio, D. S. The state-trait anxiety inventory. *Rev. Interam. Psicol. J. Psychol.* **5**, 3–4 (1971).

[10] Beck AT, Guth D, Steer RA, Ball R (1997). Screening for major depression disorders in medical inpatients with the Beck Depression Inventory for Primary Care. Behav. Res. Ther..

[11] Forte G, Favieri F, Tambelli R, Casagrande M (2020). COVID-19 pandemic in the italian population: Validation of a post-traumatic stress disorder questionnaire and prevalence of PTSD symptomatology. Int. J. Environ. Res. Public Health.

[12] Bilker WB (2012). Development of abbreviated nine-item forms of the Raven’s standard progressive matrices test. Assessment.

[13] Oswald FL, McAbee ST, Redick TS, Hambrick DZ (2014). The development of a short domain-general measure of working memory capacity. Behav. Res. Methods.

[14] Kane MJ, Bleckley MK, Conway AR, Engle RW (2001). A controlled-attention view of working-memory capacity. J. Exp. Psychol. Gen..

[15] Hutchison KA (2007). Attentional control and the relatedness proportion effect in semantic priming. J. Exp. Psychol..

[16] Luck SJ, Vogel EK (1997). The capacity of visual working memory for features and conjunctions. Nature.

[17] Shipstead Z, Lindsey DRB, Marshall RL, Engle RW (2014). The mechanisms of working memory capacity: Primary memory, secondary memory, and attention control. J. Mem. Lang..

[18] Ragland JD (2012). Relational and item-specific encoding (RISE): Task development and psychometric characteristics. Schizophr. Bull..

[19] Peterson RA (2021). Finding optimal normalizing transformations via bestNormalize. R J..

[20] Korkmaz S, Goksuluk D, Zararsiz G (2014). MVN: An R package for assessing multivariate normality. R J..

[21] Tabachnick, B. G., Fidell, L. S. & Ullman, J. B. *Using Multivariate Statistics* 6 (Pearson 2013).

[22] Ferrucci R (2021). Long-lasting cognitive abnormalities after COVID-19. Brain Sci..

[23] Almeria M, Cejudo JC, Sotoca J, Deus J, Krupinski J (2020). Cognitive profile following COVID-19 infection: Clinical predictors leading to neuropsychological impairment. Brain Behav. Immunity. Health.

[24] Herrera E (2023). Cognitive impairment in young adults with post COVID-19 syndrome. Sci. Rep..

[25] Hampshire A (2021). Cognitive deficits in people who have recovered from COVID-19. eClinicalMedicine.

[26] Becker JH (2021). Assessment of cognitive function in patients after COVID-19 infection. JAMA Netw. Open.

[27] Callan C, Ladds E, Husain L, Pattinson K, Greenhalgh T (2022). I can’t cope with multiple inputs’: A qualitative study of the lived experience of brain fog’ after COVID-19. BMJ Open.

[28] Bertuccelli M (2022). Cognitive impairment in people with previous COVID-19 infection: A scoping review. Cortex.

[29] Crivelli L (2022). Changes in cognitive functioning after COVID-19: A systematic review and meta-analysis. Alzheimer’s Dement..

[30] Biagianti B (2022). Cognitive assessment in SARS-CoV-2 patients: A systematic review. Front. Aging Neurosci..

[31] Teodoro T, Edwards MJ, Isaacs JD (2018). A unifying theory for cognitive abnormalities in functional neurological disorders, fibromyalgia and chronic fatigue syndrome: Systematic review. J. Neurol. Neurosurg. Psychiatry.

[32] Ball HA (2020). Functional cognitive disorder: Dementia’s blind spot. Brain.

[33] Perez DL (2021). Neuroimaging in functional neurological disorder: State of the field and research agenda. NeuroImage Clin..

[34] Ajčević M (2023). Cerebral hypoperfusion in post-COVID-19 cognitively impaired subjects revealed by arterial spin labeling MRI. Sci. Rep..

[35] Teodoro T, Chen J, Gelauff J, Edwards MJ (2023). Functional neurological disorder in people with long COVID: A systematic review. Eur. J. Neurol..

[36] Bhome R (2022). Metacognition in functional cognitive disorder. Brain Commun..

[37] Parker C (2021). Depression, anxiety, and acute stress disorder among patients hospitalized with COVID-19: A prospective cohort study. J. Acad. Consult. Psychiatry.

[38] Cai X (2020). Psychological distress and its correlates among COVID-19 survivors during early convalescence across age groups. Am. J. Geriatr. Psychiatry.

[39] Gouraud C (2021). Association between psychological distress, cognitive complaints, and neuropsychological status after a Severe COVID-19 episode: A cross-sectional study. Front. Psychiatry.

