# Supplementary material for: Insights into attention and memory difficulties in post-COVID syndrome using standardized neuropsychological tests and experimental cognitive tasks
Source: Sci Rep. 2024 Feb 22;14:4405. doi: 10.1038/s41598-024-54613-9 (PMC10883994; doi:10.1038/s41598-024-54613-9)
Supplement: Supplementary file 1 — Supplementary Information 1. [file 41598_2024_54613_MOESM1_ESM.pdf]

**Table S1 –** Demographical and neuropsychological data

| | | | | | MoCA[1] | PRMQ[2] | | | Digit Span[3] | | Word list[4] | | | | TMT[5] | | | SDMT[6] | Stroop[7] | | PASAT[8] | MFPT[9] | | | Fluency[10] | | Naming[11] | COVID PTSD[12] |
|---|---|---|---|---|---|---|---|---|---|---|---|---|---|---|---|---|---|---|---|---|---|---|---|---|---|---|---|---|
| ID | Sex | Age | Edu | Onset | | prospective | retrospective | total | forward | backward | immediate recall | delayed recall | non recalled | recognition | TMT-A | TMT-B | TMT-B-A | | congruent | incongruent | | unique designs | strategies | errors | phonemic | semantic | | |
| 1 | M | 60 | 10 | 3 | -1.23 | -0.37 | -0.66 | -0.56 | -0.88 | -0.57 | -0.25 | -0.32 | 0.21 | -0.67 | 0.91 | 0.32 | -0.12 | 0.30 | -0.20 | -0.92 | -0.45 | 0.39 | 0.12 | 0.23 | -1.20 | 3.83 | -3.51 | -0.20 |
| 2 | F | 48 | 13 | 6 | 0.08 | -2.61 | -2.07 | -2.53 | 0.84 | 0.44 | 2.16 | 0.99 | 0.47 | 0.11 | -0.76 | -0.46 | -0.06 | 0.18 | -0.70 | -0.08 | -0.14 | -0.27 | -0.39 | 0.23 | 0.27 | 1.92 | 0.78 | 0.87 |
| 3 | F | 48 | 16 | 12 | 0.34 | -3.63 | -2.07 | -3.07 | -0.80 | -0.37 | 0.01 | -0.67 | -0.42 | -0.50 | -0.94 | -2.68 | -2.00 | -0.28 | -0.41 | -0.26 | -1.35 | -0.85 | -0.59 | 0.23 | -1.47 | -0.32 | -1.22 | 0.50 |
| 4 | F | 43 | 13 | 2 | -0.23 | -2.41 | -1.87 | -2.31 | -0.03 | 0.44 | -1.21 | -0.22 | 0.35 | -0.76 | -0.11 | -0.30 | -0.29 | -0.05 | -0.70 | 0.42 | 0.26 | -0.13 | -0.05 | 1.08 | 1.51 | 2.38 | -0.63 | -0.51 |
| 5 | F | 48 | 12 | 3 | -0.42 | -0.98 | -2.47 | -1.87 | -0.93 | -1.54 | -1.39 | -1.08 | -0.29 | -1.57 | -1.12 | -2.75 | -2.55 | 0.05 | -1.07 | -0.76 | -0.53 | -2.11 | -0.74 | 0.08 | -0.86 | -0.24 | -1.22 | 0.31 |
| 6 | F | 52 | 13 | 1 | -0.11 | n.a. | n.a. | n.a. | 0.31 | 0.38 | -0.66 | -0.35 | 0.64 | -0.27 | 0.06 | 0.15 | 0.33 | n.a. | -0.70 | -0.85 | -0.20 | -1.81 | -0.75 | 0.23 | -1.54 | -1.37 | 0.78 | 0.12 |
| 7 | F | 64 | 11 | 6 | 0.39 | -2.20 | -1.67 | -2.09 | 0.34 | -0.15 | 0.20 | 0.81 | 0.38 | 0.14 | 0.01 | -0.48 | -0.62 | 0.49 | -0.23 | 0.25 | 0.31 | -0.70 | -0.30 | -1.05 | 0.20 | 3.54 | 0.57 | -0.01 |
| 8 | F | 60 | 13 | 5 | 0.55 | -3.43 | -1.87 | -2.85 | 0.31 | 1.99 | 1.99 | 2.67 | 0.69 | 0.32 | 0.40 | 0.35 | -0.45 | 0.62 | -0.10 | 0.86 | 0.17 | 0.95 | -0.36 | -0.20 | 0.37 | 3.28 | -0.45 | -0.64 |
| 9 | M | 57 | 11 | 8 | -0.27 | -1.39 | -0.87 | -1.22 | -0.83 | 0.29 | 0.32 | 0.22 | -0.75 | 0.14 | 0.50 | -0.21 | -0.71 | -0.30 | -0.15 | -0.70 | 1.81 | -0.24 | -0.41 | -0.34 | 0.69 | 0.03 | -0.45 | -0.95 |
| 10 | F | 40 | 13 | 5 | 0.50 | 0.24 | 1.14 | 0.75 | 0.65 | 0.94 | 1.89 | 1.29 | 0.80 | 0.11 | 0.69 | 0.62 | 0.30 | 0.07 | -0.24 | -0.26 | 1.07 | 0.28 | -0.23 | 1.08 | -0.87 | 1.76 | -0.63 | 1.06 |
| 11 | F | 52 | 17 | 6 | 0.65 | -2.61 | -1.07 | -2.31 | 2.56 | 2.17 | 1.52 | 1.06 | 0.42 | 0.34 | 0.55 | -0.51 | -1.19 | -0.46 | -0.97 | -0.22 | -0.94 | -1.13 | -0.46 | 0.08 | -1.10 | 0.52 | -1.22 | 0.87 |
| 12 | M | 47 | 16 | 4 | -0.18 | -2.20 | -0.46 | -1.43 | -0.80 | -0.37 | -1.58 | -2.18 | -2.25 | -3.24 | -6.05 | -7.75 | -3.96 | -1.73 | -1.28 | -2.14 | -1.95 | -0.75 | -0.88 | 0.65 | -0.69 | -0.90 | -3.22 | 0.94 |
| 13 | F | 54 | 17 | 7 | 0.69 | -0.17 | -0.26 | -0.23 | 2.56 | 0.55 | 1.29 | 1.89 | 1.31 | 0.34 | -0.33 | -0.27 | -0.16 | 0.10 | 0.17 | 0.82 | -0.66 | -0.07 | -0.43 | 1.08 | 0.33 | 1.40 | -1.22 | 0.50 |
| 14 | M | 61 | 16 | 7 | 0.32 | -1.80 | -1.67 | -1.87 | 2.72 | 1.26 | 1.57 | 2.48 | 0.69 | 0.40 | -1.53 | 0.50 | 1.15 | 0.33 | -0.72 | 0.59 | 1.17 | -1.10 | -0.54 | 0.51 | 1.97 | 2.47 | -1.47 | -2.40 |
| 15 | F | 58 | 10 | 12 | 0.77 | -2.00 | -1.47 | -1.87 | -0.88 | -0.57 | -0.86 | -1.37 | 0.35 | -3.01 | -0.86 | -0.67 | -0.31 | -0.96 | -1.01 | -0.61 | -2.10 | -1.71 | -0.05 | 1.08 | -0.55 | 0.98 | 0.57 | -1.39 |
| 16 | F | 52 | 17 | 4 | 0.65 | 0.44 | 1.34 | 0.97 | 0.46 | 0.55 | -2.08 | -1.02 | 1.31 | -0.07 | -0.91 | -3.73 | -4.94 | -0.74 | -0.80 | -0.09 | -3.45 | -2.28 | -0.74 | -1.19 | 0.61 | -0.61 | 0.78 | -1.83 |
| 17 | M | 56 | 12 | 9 | -0.04 | -2.61 | -1.87 | -2.42 | 0.26 | 0.34 | -0.55 | -0.28 | 1.17 | -0.34 | 0.63 | -0.38 | -1.15 | 0.72 | 0.62 | 0.06 | 1.03 | -1.29 | -0.07 | -0.34 | 1.25 | 1.32 | -3.51 | -0.07 |
| 18 | M | 53 | 13 | 9 | 1.18 | -0.17 | -0.46 | -0.34 | 2.41 | 2.80 | 0.95 | 1.74 | 1.31 | 0.44 | 0.72 | 0.46 | 0.29 | 1.18 | 0.59 | 0.91 | 2.11 | 1.64 | -0.04 | -0.06 | 1.53 | 2.58 | -3.22 | -0.20 |
| 19 | F | 56 | 13 | 10 | 0.98 | -1.59 | 0.34 | -0.67 | 1.36 | 1.19 | 1.14 | 0.90 | 0.35 | 0.56 | 0.46 | 0.35 | 0.21 | 0.00 | -0.28 | -0.25 | 0.03 | -1.29 | -0.41 | 0.37 | -0.01 | 1.51 | -0.45 | 0.43 |
| 20 | F | 55 | 12 | 14 | 0.96 | -3.02 | -3.48 | -3.51 | 1.32 | -0.47 | 2.34 | 2.22 | 1.31 | 0.62 | 0.72 | -0.52 | -1.47 | -0.16 | -1.18 | -0.09 | 0.87 | 1.68 | -0.08 | -0.34 | 1.05 | 2.14 | -4.53 | -0.39 |
| 21 | F | 66 | 13 | 6 | -0.32 | -2.20 | -1.47 | n.a. | 0.43 | -0.02 | -0.31 | -0.53 | 0.27 | -0.96 | -2.93 | -4.10 | -3.56 | -1.25 | -1.80 | -1.29 | -1.47 | -1.58 | -0.55 | 1.08 | -0.30 | -0.25 | 0.32 | -0.57 |
| 22 | F | 47 | 17 | 9 | -0.69 | 0.85 | 1.74 | 1.41 | -0.77 | -0.32 | 0.19 | 0.80 | 0.80 | 0.32 | -1.39 | -4.34 | -3.31 | 0.24 | 0.29 | 0.03 | -0.41 | -0.41 | -0.95 | 0.65 | 0.71 | 0.42 | -1.22 | -0.13 |
| 23 | F | 59 | 13 | 12 | 0.28 | -2.41 | -1.67 | -2.20 | 2.41 | 1.99 | 0.24 | 0.07 | 0.20 | -0.27 | 0.32 | -0.47 | -1.03 | 0.10 | 0.81 | -0.16 | -0.43 | -0.44 | -0.09 | 1.08 | 2.56 | 4.10 | -0.45 | -1.58 |
| 24 | F | 53 | 16 | 21 | 0.93 | -2.00 | -2.07 | -2.20 | 1.48 | -0.29 | 2.38 | 2.37 | 1.31 | 0.40 | 0.35 | -0.46 | -0.97 | -0.27 | -0.74 | -0.01 | -0.55 | -1.00 | -0.52 | 0.51 | 0.34 | 1.19 | -1.22 | -0.51 |
| 25 | M | 64 | 10 | 13 | 0.39 | -2.20 | -1.67 | -2.09 | 0.29 | 0.88 | -0.59 | -1.92 | -1.74 | 0.10 | -2.42 | -2.37 | -1.62 | -0.14 | -0.60 | -0.29 | -0.45 | -1.27 | -0.38 | 0.23 | 0.13 | 2.22 | -1.47 | -2.46 |
| 26 | F | 59 | 13 | 12 | 0.02 | -1.19 | -0.26 | -0.78 | 0.31 | 1.19 | 0.57 | 0.90 | 0.80 | 0.56 | 0.77 | -0.18 | -0.91 | 0.52 | 0.72 | 0.09 | -0.32 | 0.59 | -0.51 | 1.08 | -0.01 | n.a. | -2.49 | -1.46 |
| 27 | F | 47 | 13 | 6 | 0.33 | -3.22 | -2.07 | -2.85 | 0.84 | 1.40 | -0.40 | 0.08 | 0.39 | -0.33 | -0.54 | -0.84 | -0.65 | -1.20 | -1.90 | -0.84 | -1.65 | -1.09 | -0.68 | 1.08 | -1.16 | -0.07 | -1.22 | -0.39 |
| 28 | F | 51 | 8 | 12 | -1.91 | -3.83 | -3.28 | -3.84 | -0.98 | -1.49 | -0.79 | -1.21 | -1.36 | 0.47 | 0.26 | -0.02 | -0.07 | -0.52 | 0.15 | -1.63 | -3.20 | -1.36 | -0.29 | -7.71 | -0.25 | 0.17 | -1.22 | -0.70 |
| 29 | F | 39 | 17 | 10 | 1.00 | -4.04 | -2.87 | -3.73 | -0.13 | -0.78 | -0.41 | 0.27 | 1.03 | -0.78 | -0.03 | -0.02 | -0.01 | -0.15 | -0.36 | 0.48 | 0.39 | -0.54 | -0.79 | -0.48 | 1.28 | 0.82 | -0.63 | 0.50 |
| 30 | M | 55 | 13 | 11 | 0.20 | -0.78 | -0.87 | -0.89 | 1.36 | 1.19 | -0.40 | -0.76 | -0.16 | -0.40 | 0.55 | 0.10 | -0.19 | 0.32 | 0.21 | 0.10 | 0.61 | -0.27 | -0.29 | -5.72 | 0.48 | 1.21 | -0.45 | 0.05 |
| 31 | F | 56 | 16 | 12 | 0.73 | -1.19 | -0.46 | -0.89 | 0.42 | 0.51 | 0.02 | 0.70 | -0.03 | -0.02 | -0.33 | -0.81 | -0.97 | -0.18 | -0.93 | 0.07 | 0.72 | 0.54 | -0.34 | 1.08 | -0.04 | -0.72 | 0.57 | 0.62 |

*Abbreviations*: ID = identification code, M = male, F = female, Edu = years of education, Onset = months from onset of illness to assessment, n.a. = data not available.


[1] Aiello, E. N. et al. The Montreal Cognitive Assessment (MoCA): updated norms and psychometric insights into adaptive testing from healthy individuals in Northern Italy. Aging Clin. Exp. Res. 34, 375–382 (2022).

[2] Crawford, J. R., Smith, G., Maylor, E. A., Della Sala, S. & Logie, R. H. The Prospective and Retrospective Memory Questionnaire (PRMQ): Normative data and latent structure in a large non-clinical sample. Memory 11, 261–275 (2003).

[3] Monaco, M., Costa, A., Caltagirone, C. & Carlesimo, G. A. Forward and backward span for verbal and visuo-spatial data: Standardization and normative data from an Italian adult population. Neurol. Sci. 34, 749–754 (2013).

[4] Mauri, M. et al. Standardizzazione di due nuovi test di memoria: apprendimento di liste di parole correlate e non correlate semanticamente. Arch. di Psicol. Neurol. e Psichiatr. 58, 621–645 (1997).

[5] Siciliano, M. *et al*. Regression-based normative data and equivalent scores for Trail Making Test (TMT): an updated Italian normative study. *Neurol. Sci*. **40**, 469–477 (2019).

[6] Nocentini, U., Giordano, A., Di Vincenzo, S., Panella, M. & Pasqualetti, P. The symbol digit modalities test - Oral version: Italian normative data. Funct. Neurol. 21, 93–96 (2006).

[7] Brugnolo, A. *et al*. An updated Italian normative dataset for the Stroop color word test (SCWT). *Neurol. Sci*. **37**, 365–372 (2016).

[8] Saetti, M. C. *et al*. The Paced Auditory Serial Addition Task (PASAT): normative data for the Italian population. *Neuropsychol. Trends* 65–82 (2021).

[9] Cattelani, R., Dal Sasso, F., Corsini, D. & Posteraro, L. The Modified Five-Point Test: Normative data for a sample of Italian healthy adults aged 16-60. *Neurol. Sci*. **32**, 595–601 (2011)

[10] Costa, A. *et al*. Standardization and normative data obtained in the Italian population for a new verbal fluency instrument, the phonemic/semantic alternate fluency test. *Neurol. Sci*. **35**, 365–372 (2014).

[11] Catricalà, E. *et al*. An Italian battery for the assessment of semantic memory disorders. *Neurol. Sci*. **34**, 985–993 (2013).

[12] Forte, G., Favieri, F., Tambelli, R. & Casagrande, M. COVID-19 pandemic in the italian population: Validation of a post-traumatic stress disorder questionnaire and prevalence of PTSD symptomatology. Int. J. Environ. Res. Public Health 17, 1–16 (2020).
