# Supplementary material for: Insights into attention and memory difficulties in post-COVID syndrome using standardized neuropsychological tests and experimental cognitive tasks
Source: Sci Rep. 2024 Feb 22;14:4405. doi: 10.1038/s41598-024-54613-9 (PMC10883994; doi:10.1038/s41598-024-54613-9)
Supplement: Supplementary file 2 — Supplementary Information 2. [file 41598_2024_54613_MOESM2_ESM.docx]

**Supplementary material**

**Detailed computerized task battery description**

**Working memory capacity.** To evaluate working memory capacity, we employed shortened versions of the Operation and Symmetry span tasks. In the Operation Span task, participants were presented with a series of arithmetic operations and asked to judge their validity, while in the Symmetry Span task, participants were presented with 8x8 matrices of black and white squares and had to determine their vertical axis symmetry. After each arithmetic operation or matrix presentation, participants were required to remember a specific element (a letter in the Operation Span task and a red square in the Symmetry Span task) for later recall. The Operation Span task had set sizes ranging from 4 to 6, administered two times, resulting in 30 operation-storage pairs. On the other hand, the Symmetry Span task had set sizes ranging from 3 to 5, also administered two times, resulting in 24 symmetry-storage pairs. For both tasks, the performance measure was the partial span score, which was calculated as the total number of items accurately recalled in their correct serial position.

**Antisaccade task.** Participants were presented with a central fixation cross that appeared for a variable duration ranging from 2,000 ms to 3,000 ms, followed by a 300 ms alerting tone. After the tone, an asterisk appeared for 300 ms to the left or right of the center, followed by a letter (Q or O) displayed on the opposite side of the screen for 200 ms. Both the asterisk and letter were masked for 500 ms. The participants' task was to disregard the asterisk and shift their attention to the opposite side of the screen to identify the target letter. They had unlimited time to respond by pressing the corresponding key on the keyboard and were provided with feedback on their accuracy displayed for 500 ms, followed by a blank intertrial interval lasting 1,000 ms. Each participant completed a total of 72 trials, and the dependent measure was the number of correctly identified target letters.

**Selective visual arrays.** Participants viewed a display of blue and red rectangles with different orientations for 250 ms and were instructed to attend to either the red or blue rectangles based on a preceding cue presented for 300 ms. After a brief delay of 900 ms, the display reappeared, showing only the target rectangles (red or blue). One of the target rectangles, indicated by a white dot, randomly changed its orientation on half of the trials. Participants were required to indicate whether the rectangle with the white dot had changed its orientation from the initial presentation. A total of 80 trials were administered, with each trial presenting an array of either five or seven rectangles per color. A capacity score (*k*) was calculated for each participant using the single probe correction [1] (*k*= N × (hits + correct rejections − 1), where N represents the size of the array) and used as the dependent variable.

**Adaptive Flanker task (deadline version).** In the present study, we adopted a modified version of the arrow flanker task in which an adaptive procedure was used to estimate the participant’s performance. They were shown an arrow at the center of the screen pointing left or right, along with two surrounding arrows on each side that either matched the direction of the central arrow (congruent trial: e.g., 🡨🡨🡨🡨🡨) or pointed in the opposite direction (incongruent trial: e.g., 🡪🡪🡨🡪🡪). The participants were instructed to indicate the direction of the central arrow by pressing the Z key for left or the "/" key for right. The keys were labeled with the words LEFT and RIGHT to aid in response mapping. A total of 324 trials were administered across 18 blocks, with each block consisting of 18 trials. In each trial, the arrows remained on the screen until the response deadline, after which a loud beep indicated the end of the trial. This response deadline varied based on the participant's accuracy within each block. If the participant responded correctly on at least 15 out of 18 trials, the deadline decreased by 90 ms; otherwise, it increased by 270 ms. From the seventh block onwards, the deadline changed by 30 ms (decrease) or 90 ms (increase). The initial block had a response deadline of 1,050 ms. In each block, 12 congruent and 6 incongruent trials were presented in random order, with an interstimulus interval (ISI) ranging from 400 ms to 700 ms. Accuracy on both trial types determined whether the response deadline increased or decreased in the next block. RTs and accuracy on incongruent trials were used as dependent variables.

**Long-term memory.** The stimuli used in the Relational and Item Specific Encoding task consisted of visual object representations selected from a standardized corpus of color photographs. There were three test forms, and each included a list of 36 items for item-specific encoding, 36 items for relational encoding, and 72 new items for recognition testing. Participants first performed two incidental encoding tasks. In the item-specific encoding task, participants viewed 36 stimuli, presented for 2000 ms each with a 1000 ms ISI. They were instructed to indicate whether the objects were "living" using a two-button yes/no response. In the Relational Encoding task, participants viewed 18 pairs of visual objects, presented for 4000 ms each with a 1000 ms ISI. They were instructed to indicate whether one object could fit inside the other using a two-button yes/no response. The two encoding tasks were alternated between them presenting 3 item-encoding blocks of 12 trials each and 3 relational-encoding blocks of 6 trials each. After the encoding phase, two retrieval tasks were administered. In the item recognition task, all 72 studied objects (36 item-specific and 36 relational targets) were presented randomly mixed with 72 new items. In each trial, participants were prompted to indicate whether each item was "old" or "new" by using a two-button response and to provide a confidence rating using a 3-button scale (3 = high, 2 = medium, 1 = low). In the associative recognition task, the 18 object pairs studied during relational encoding were presented randomly, mixed with 18 rearranged object pairs originally not paired together (e.g., an item from trial 1 paired with the item from trial 12). Participants were prompted to indicate whether the items in each pair had been presented "together" by using a two-button yes/no response. For both tasks, the dependent variable was the recognition memory discriminability index (d´), computed by subtracting the z scored false alarm rate (i.e., proportion of new/rearranged items erroneously identified as old/unvaried) from the z scored hit rate (i.e., proportion of old/unvaried items correctly identified).

1. Cowan, N. *et al.* On the capacity of attention: its estimation and its role in working memory and cognitive aptitudes. *Cogn. Psychol.* **51,** 42–100 (2005).
